# Recombinant protein expression in biofilms

**DOI:** 10.3934/microbiol.2019.3.232

**Published:** 2019-08-27

**Authors:** Alexandra Soares, Ana Azevedo, Luciana C. Gomes, Filipe J. Mergulhão

**Affiliations:** Laboratory for Process Engineering, Environment, Biotechnology and Energy, Faculty of Engineering, University of Porto, Rua Dr. Roberto Frias, 4200-465 Porto, Portugal

**Keywords:** recombinant protein, green fluorescent protein, biofilm, bacteria, *Escherichia coli*, filamentous fungi

## Abstract

Biofilm research is usually focused on the prevention or control of biofilm formation. Recently, the significance of the biofilm mode of growth in biotechnological applications received increased attention. Since biofilm reactors show many advantages over suspended cell reactors, especially in their higher biomass density and operational stability, bacterial biofilms have emerged as an interesting approach for the expression of specific proteins. Despite the potential of biofilm systems, recombinant protein production using biofilms has been scarcely investigated for the past 25 years. Our group has demonstrated that *E. coli* biofilms were able to produce a model recombinant protein, the enhanced green fluorescent protein (eGFP), at much higher levels than their planktonic counterparts. Even without optimization of cultivation conditions, an attractive productivity was obtained, indicating that biofilm cultures can be used as an alternative form of high cell density cultivation (HCDC). *E. coli* remains one of the favorite hosts for recombinant protein production and it has been successfully used in metabolic engineering for the synthesis of high value products. This review presents the advantages and concerns of using biofilms for the production of recombinant proteins and summarizes the different biofilm systems which have been described for this purpose. The relative advantages and disadvantages of the four microbial hosts tested for recombinant protein production in biofilms (two bacteria and two filamentous fungi) are also discussed.

## 1. Introduction

Recombinant proteins are synthesized in a host cell which is usually of a different species from the source of the DNA encoding them [1] and in that case they can be called heterologous proteins. Recombinant proteins have wide applications in medicine, research and biotechnology. With the development of recombinant protein methodology, it is possible to clone genes encoding proteins from different organisms and express them in other organisms at much higher levels than those naturally achieved [1],[2], leaving behind the necessity of huge amounts of animal and plant tissues or volumes of biological fluids [3].

To obtain recombinant proteins, the gene encoding the protein is isolated and introduced into an expression vector, afterwards it is transformed into the chosen host system [1],[3]. An important step in recombinant protein production is the choice of the ideal expression system. A large number of protein expression hosts are available, such as bacteria, yeast, filamentous fungi, and mammalian, plant and insect cells [4],[5]. It is also important to select the most suitable expression vector, which is composed by a set of genetic elements that affect both transcriptional and translational steps of protein production, namely the origin of replication, promoter, ribosome binding site, start codon, transcriptional terminator, and selective marker [3].

Recombinant proteins have been mostly produced in suspended cultures. The insertion of the gene of interest into a multicopy plasmid may result in high recombinant protein expression [1]. Nevertheless, this may impose a metabolic burden on the host cell due to the energy and metabolites needed for the replication of plasmid DNA and synthesis of recombinant proteins [1],[6]. In planktonic cells, this added metabolic burden may decrease the cellular growth rates and biomass yield, besides affecting the yield and activity of the desired protein [6]. In order to overcome these problems, a strategy based on the use of biofilm systems has been studied. Microbial biofilms are communities of microorganisms, single or multispecies, attached to surfaces which are embedded in a self-produced matrix of extracellular polymers [7]–[9]. Biofilms are usually known for their negative effects on health and industrial sectors as they can cause diseases, equipment corrosion, local clogging, heat transfer resistance and product contamination in food processing environments [7],[10]. However, biofilms have beneficial use in wastewater treatment [11] and are being tested for the production of solvents, organic acids and enzymes [12]–[14]. The biological organization of biofilms provides them with many advantages over the suspended cells, including high cell density and protection against hostile conditions [15]. Furthermore, the presence of expression vectors in the sessile cells has shown to increase biofilm formation and lead to higher production of recombinant proteins compared to planktonic cells [16]–[19].

This review will focus on the production of recombinant proteins by biofilms. The main studies using biofilm systems for the production of recombinant proteins will be explored, as well as the differences between producing recombinant proteins in suspended and biofilm cultures. The advantages and disadvantages of using different microbial hosts in the production of recombinant proteins, namely *Escherichia coli*, *Bacillus subtilis*, *Aspergillus niger* and *Aspergillus oryzae*, will also be addressed.

## 2. Recombinant protein expression in suspended and biofilm cultures

Biofilm systems have many advantages when compared with suspended growth systems, especially in their higher biomass density and operation stability. Biofilm reactors are able to retain 5 to 10 times more biomass per unit volume of reactor [15], thus increasing the production rates and reducing the risk of cell washout when operating at high dilution rates during continuous fermentation. Furthermore, biofilms provide a protective environment to the cells as the extracellular matrix protects them against extreme conditions of pH and temperature, contaminations, hydraulic shock, antibiotics and toxic substances [15]. Another advantage of biofilm cultures is that cells can be easily separated from the liquid by sedimentation or filtration, resulting in more efficient downstream processes [13].

Recombinant protein production in biofilms was first described 25 years ago by Huang et al. [20]–[22], who showed that the maximum β-galactosidase concentration obtained in *E. coli* DH5α suspended cells was higher than that obtained in biofilm cells (0.47 vs. 0.12 pg/cell) [21]. On the other hand, the probability of plasmid loss in a biofilm culture was greater than that observed in suspended batch cultures [21]. These results suggested that the production of extracellular polymeric substances (EPS) by biofilm cells may compete with plasmid maintenance/replication and expression of an heterologous plasmid-encoded protein for metabolic intermediates and energy sources, a competition that suspended cells do not experience [21]. About 15 years later, O'Connell and colleagues [17] contradicted this previous work by demonstrating that the *E. coli* biofilm environment enhanced the heterologous protein production when compared to planktonic cells. This study was carried out with *E. coli* ATCC 33456 pEGFP, a strain that formed a robust biofilm and harbored a high copy number modified pUC vector encoding enhanced green fluorescent protein (eGFP). Additionally, cultivation of *E. coli* as a biofilm was found to have a beneficial effect on high copy number plasmid maintenance compared to chemostats [17], which increased the gene dosage and may have contributed to the fact that 90% or more of biofilm cells produced significant levels of eGFP after 6 days, even in the absence of selective pressure. The reason for the enhanced plasmid maintenance in biofilms is that sessile cells tend to grow more slowly than their planktonic counterparts [23], leading to fewer cell divisions and consequently less plasmid partitioning. With infrequent cell division, less energy is directed towards replication, reducing the metabolic burden of plasmid maintenance on the cell. More recently, our research group assessed the potential of *E. coli* JM109(DE3) biofilms in the expression of the model protein eGFP from a pET-based vector [16],[18],[24],[25]. It was found that the specific protein production from *E. coli* biofilm cells was about 30 fold higher than in planktonic state (5.8 vs. 0.18 fg/cell) [16]. When lysogeny broth (LB) was tested, which is a common culture medium used for recombinant protein expression with the pET system, the difference between the specific eGFP production of biofilms and suspended cells decreased to 10 times (12 and 1.2 fg/cell in biofilm and cell suspension, respectively) [18],[25]. Furthermore, the percentage of planktonic eGFP-expressing cells oscillated around 5%, whereas for biofilms eGFP-expressing cells represented 21% of the total cell population [18]. The higher productivity of *E. coli* biofilms is probably related to their higher potential in maintaining the plasmid within the cells. In fact, in planktonic cells, the frequency of plasmid-containing bacteria was on average 0.33, while in the biofilm this parameter rose to approximately 0.90 [18].

In addition to *E. coli*, another bacteria - *Bacillus subtilis* - was studied for recombinant protein production in biofilms. The biofilm was reported as a new fermentation technique wherein the iturin A concentration almost doubled that obtained in the submerged culture [26]. This result was explained by the fact that biofilm cells remain in their metabolically active state for a longer period of time and maximize nutrient utilization [26].

The comparative analysis of classical fermentation and biofilm reactors for the production of recombinant proteins was also performed for the filamentous microorganisms *A. niger* and *A. oryzae* [27],[28]. Talabardon and Yang [28] cultured a recombinant strain of *A. niger* containing the gene coding for GLA-GFP (glucoamylase-green fluorescent protein) fusion protein on cotton cloth in two different biofilm reactor configurations. They revealed that the biofilms formed in these conditions produced 10 fold more fusion protein than free-living pellets [28]. Zune et al. [27] also showed that the fluorescence signal measured from the same fusion protein produced in *A. oryzae* biofilm reactors was two times higher than that obtained in a tank operating at a low stirrer rate. In the case of filamentous microorganisms, the immobilization in biofilm reactors leads to decreased medium viscosity and consequently to an enhanced nutrient and oxygen transfer [29],[30], which in turn may have increased the product yield. At the same time, the internal structure of fungal biofilms comprises channels in the hyphal layers that allow fluid circulation and promote a better mass transfer in comparison with the more compact structure of the pellets found in submerged culture [31]. Another reason for the higher recombinant protein levels found in *Aspergillus* biofilm cultures is the low protease secretion [32],[33].

Although biofilm reactors show several advantages for recombinant protein production over planktonic cultures, there are some limitations, particularly in the diffusion of substrates to the bacterial cells. Usually bacterial biofilms contain multiple layers of cells and their thickness may vary from a few to many µm. In order for the cells to be active in the production of value-added compounds, nutrients and substrate must diffuse/penetrate to the inner layers of the biofilm. However, it is known that sometimes the nutrients and substrate are used up by the outer cell layers before they reach the innermost layers [14],[24]. According to a previous work from our research group [24], eGFP-producing biofilms are highly heterogeneous, with the cells actively producing the recombinant protein restricted to the top layer of the biofilm. This is probably due to the lack of oxygen penetration inside the biofilm, which is necessary for eGFP maturation, and/or mass transfer limitation of nutrients. Hence, the operating conditions should be optimized in order to obtain a porous biofilm, thus facilitating the access of the bottom layer to oxygen and fresh nutrients, which could then be shifted to a productive state [14],[24],[34]. Nevertheless, some authors have shown that diffusion limitations are not always harmful to recombinant protein expression in biofilms [21],[35]. Mass transfer limitations of nutrients may cause lower cell growth rates inside the biofilm, thereby contributing to increased plasmid stability [21],[35].

An additional drawback of recombinant protein production in biofilm cultures may come from the toxicity of the protein, which arises when it performs an unnecessary and detrimental function in the host cell [3]. Another aspect to consider is the location of the recombinant protein since the biofilm approach is more appropriate if the protein is released from the biofilm and can be captured afterwards from the culture medium [36]. If the recombinant protein is located intracellularly, a “milking” process must be established to operate the reactor in a continuous mode. This is a challenging task, in particular with regard to process engineering and economics of the process.

**Figure 1.**
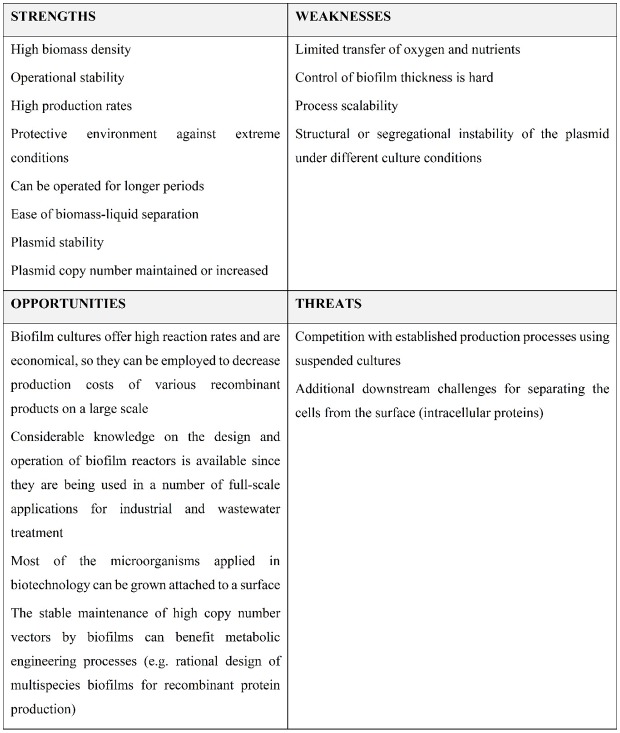
SWOT analysis of biofilm cultures for recombinant protein production.

The Strengths, Weaknesses, Opportunities and Threats (SWOT) analysis of the application of biofilm reactors in recombinant protein production is presented in Figure 1.

## 3. Application of biofilms in recombinant protein production

The biofilm may provide distinct characteristics for the production of valuable compounds such as recombinant proteins [16],[17]. Despite the potential of this expressing system, recombinant protein production using biofilms has been scarcely investigated during the last decades. To the best of our knowledge, there are only seven studies from independent groups on this topic. The published work is summarized in Table 1.

The expression of recombinant proteins in biofilms was first reported by Huang et al. [20],[21],[37], who have studied the production of β-galactosidase in *E. coli* DH5α cells carrying a plasmid containing the *tac* promoter. These authors have studied the plasmid retention and expression in both suspended and biofilm cells growing in a parallel-plate flow cell (PPFC). During the course of the experiments, they found that the cell accumulation rates decreased with increasing induction levels for both suspended and biofilm cells [21]. Furthermore, the production of β-galactosidase in both types of *E. coli* cells increased with induction (no basal expression was detected). Three different IPTG concentrations (0.17, 0.34 and 0.51 mM) were tested to determine the optimal inducer concentration. In biofilm cells, different IPTG concentrations did not affect the maximum β-galactosidase concentration obtained 24 h after induction (about 0.1 pg/cell) [21]. Huang et al. [37] also shown that the concentration of nitrogen and carbon in the growth medium affected biofilm formation and plasmid stability. When the carbon/nitrogen ratio on the nutrient supply increased, the biofilm cell density decreased, while the probability of plasmid loss increased [37]. Additionally, it was shown that the synthesis rates of β-galactosidase mRNA were higher at higher expression levels, increasing 4 fold under 0.17 mM IPTG, and almost 12 fold under 0.34 and 0.51 mM IPTG. However, the β-galactosidase production did not increase at the same proportion of mRNA synthesis rate. It is possible that the synthesized mRNA was much less stable at high expression levels [20].

**Table 1.** Overview of the published work on the production of recombinant proteins in biofilms.

Host	Recombinant protein	Cultivation conditions	Recombinant protein production	Reference
*E. coli* DH5α	β-galactosidase	PPFC	0.08–0.12 pg/cell	[20],[21],[37]
		Glass slides		
		37 °C		
		Supplemented M9 minimal medium		
*E. coli* ATCC 33456	eGFP	PPFC	0.01–0.16 g/L	[17]
		Cover glass		
		37 °C		
		LB medium		
		Laminar flow		
*E. coli* JM109(DE3)	eGFP	Flow cell system	5.8 fg/cell and 0.22 g/L for DM supplemented with 20 µg/mL kanamycin 12 fg/cell for LB supplemented with 20 µg/mL kanamycin	[16],[18],[24],[25]
		PVC surfaces		
		30 °C		
		DM and LB media		
		Turbulent flow		
*Bacillus subtilis*	iturin A	24-well plates	0.6 g/L	[26]
		28 °C		
		LB medium		
*Bacillus subtilis*	mCherry, EgTrp and EgA31 (part of the TasA-mCherry, TasA-EgTrp and TasA-EgA31 fusion proteins, respectively)	Well plates with 22 mm^2^ surface area	n.a.	[38]
		30 °C		
		MSgg medium		
*Aspergillus niger*	GFP (part of the GLA-GFP fusion protein)	Static and RFB reactor	0.78 g/L	[28]
		Cotton cloth attached to a stainless steel cylinder		
		Modified Vogel's medium		
		25 °C		
*Aspergillus oryzae*	GFP (part of the GLA-GFP fusion protein)	Reactor based on a stainless steel structured packing	n.a.	[27]
		30 °C		

*Notes*: n.a: not available. *Abbreviations:* PPFC: parallel-plate flow cell; RFB: rotating fibrous bed; LB: lysogeny broth; DM: diluted medium; eGFP: enhanced green fluorescent protein; TasA-EgTrp: TasA-tropomyosin peptide; TasA-EgA31: TasA-paramyosin peptide; GLA-GFP: glucoamylase-green fluorescent protein; PVC: polyvinyl chloride.

In 2007, O'Connell et al. [17] described the first system for high level heterologous protein production in *E. coli* biofilm cells. These authors used an *E. coli* strain containing the plasmid pEGFP to investigate the production of eGFP in a chemostat (planktonic cells) and in a PPFC reactor (biofilm cells). They detected different populations of sessile cells: strongly producing cells (capable of producing 0.16 g/L of eGFP), moderately producing cells (capable of producing 0.01 g/L of eGFP) and non-producing cells. In contrast with Huang et al. [20],[21], the results of this work indicated that the biofilm environment enhanced both plasmid maintenance and heterologous protein concentration when compared to planktonic cells [17]. This study also found that the addition of low antibiotic concentrations to biofilm populations increased these beneficial effects, enabling them to obtain 60% of strongly producing cells [17].

**Figure 2.**
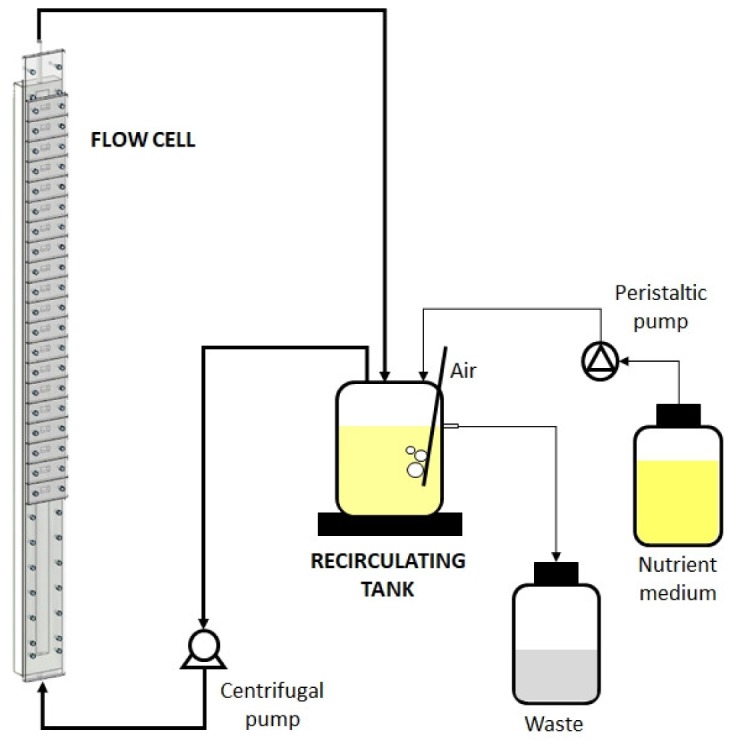
Schematic representation of the biofilm producing system.

**Figure 3.**
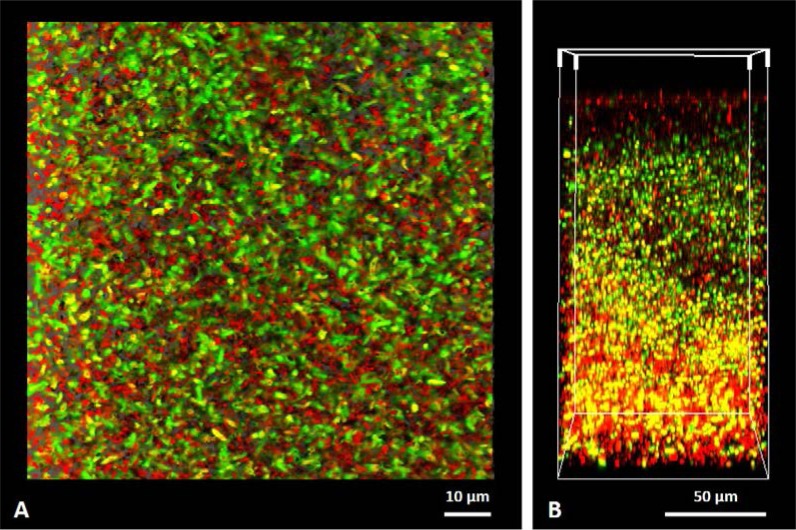
Spatial heterogeneity of a 11-day-old biofilm formed by *E. coli* JM109(DE3) expressing the recombinant protein eGFP: (A) top view and (B) three-dimensional view of the confocal laser scanning microscopy (CLSM) images. The eGFP-expressing cells are labelled in green and the non-expressing cells are countermarked in red with Syto61.

The diluted medium (mainly composed by 0.55 g/L glucose, 0.25 g/L peptone and 0.125 g/L yeast extract) was described as a good medium for biofilm formation [43], whereas LB is frequently used for the expression of recombinant proteins and provides abundant carbon and nitrogen (10 g/L tryptone and 5 g/L yeast extract) [46]. This study reported a 2 fold increase in the eGFP production of biofilms developed in LB when compared to what was obtained in DM [25]. Regarding the effect of antibiotic concentration, the increase from 20 to 30 µg/mL had no effect on the amount of recombinant protein produced by the biofilm [25]. Thus, among the tested conditions, LB supplemented with 20 µg/mL of kanamycin seemed to be the most advantageous medium to obtain the highest specific eGFP production in *E. coli* biofilms [25]. Our research group also examined the eGFP expression during biofilm development at the single-cell scale, showing that the biofilm population became increasingly heterogeneous over the course of the experiment [24]. Three different types of biofilms were observed: one with a homogeneous population (between days 3 and 5), a second with a moderately homogeneous population (between days 6 and 8), and the third with a strongly heterogeneous population (between days 9 and 11). These results are consistent with those obtained by O'Connell et al. [17], who studied the dynamics of protein fluorescence during biofilm development. Moreover, by confocal microscopic analysis (Figure 3), we also showed that the majority of the eGFP-expressing cells were located in the liquid interface of the biofilm. It is known that the spatial organization of biofilms can affect the diffusion of oxygen and nutrients, resulting in heterogeneous populations in terms of metabolic and phenotypic behaviors [47],[48]. Lenz et al. [49] observed a spatially non-uniform pattern of GFP expression in *Pseudomonas aeruginosa* biofilms. These authors demonstrated that the amount of GFP mRNA was higher in the external layers of the biofilm, which is associated with the zone of active GFP fluorescence visualized at the top of the biofilm [49]. This is probably related with the higher access of sessile cells to the oxygen required to the final stage of protein folding [50].

The production of recombinant proteins in biofilms of the Gram-positive bacterium *Bacillus subtilis* has also been studied [26],[38]. Rahman et al. [26] used a transformant strain of *B. subtilis* 168, containing wild *sfp*, the *itu* operon and *degQ* for the production of the lipopeptide iturin A. These authors reported an iturin A concentration in the biofilm of approximately 0.6 g/L in steady state conditions, which is higher than the concentration of 0.4 g/L obtained in the cell suspension [26]. Later on, Vogt et al. [38] developed a strategy based on the TasA protein for the display of a heterologous protein within the *B. subtilis* biofilm. They engineered a fusion protein with TasA and the red fluorescent protein mCherry, and showed that this fusion protein was abundant and homogeneously distributed within the biofilm. TasA is one of the crucial matrix proteins responsible for biofilm formation and is dependent on the tapA-sipW-tasA operon [51]. The same authors also produced fusion proteins of TasA with *Echinococus granulosus* antigenic peptides, paramyosin and tropomyosin [38]. The results showed that the antigens were expressed and could be efficiently located in the biofilm matrix. Additionally, it was demonstrated that the spores produced by the recombinant strain were physically and morphologically identical to the wild-type strain, which could be an excellent strategy to preserve the integrity of heterologous proteins and facilitate their transport to the desired location [38].

In 2005, Talabardon and Yang [28] used a recombinant *A. niger* strain containing a gene encoding the glucoamylase-GFP (GLA-GFP) fusion protein to study the GFP and glucoamylase protein production in suspension and immobilized biofilm cells. They used different culture systems: free-cell cultures in conventional stirred-tank bioreactors grown in pellet form and cells immobilized in cotton cloth grown in a mycelial form in a rotating fibrous bed (RFB), to study the influence of the fungal morphology in the expression of the fusion protein. These authors found that the amount of glucoamylase and GFP produced by the immobilized cells of *A. niger* was 0.8 g/L, about 10 times more than in suspended cells. Later, Zune et al. [27] used a tank with a metal structured packing as a fungal biofilm reactor for the production of the GLA-GFP fusion protein by *A. oryzae*. The fluorescence measurements indicated that protein production in the biofilm reactor was similar to that of suspended cell cultures under higher shear stress conditions. However, the western blot analysis revealed that the band corresponding to the fusion protein in submerged conditions was thicker than that under biofilm conditions. This result could be explained by the operating conditions in the submerged reactor, namely the acid pH which could promote biomass leakage and affect the quality of the protein. Based on the results obtained in the shake flasks, the authors investigated the production of the fusion protein in two different configurations of the biofilm bioreactor, one with full immersion of the fungal biofilm in the liquid medium and other with periodic immersion of the biofilm [27]. Although both biofilm reactors reached satisfactory protein productivities, a 2-D electrophoresis analysis indicated that the quality of the fusion protein was higher in the fully immersed bioreactor [27].

## 4. Hosts for recombinant protein expression in biofilms

A myriad of organisms (or their derivatives) can be chosen as hosts for recombinant protein production. The list includes bacteria, yeast, filamentous fungi, mammalian, plant and insect cells or transgenic organisms (plants and animals) [52]. Although there is a lot to choose from, the selection of the most appropriate expression host is often limited to a few options given the intrinsic characteristics of the protein and the process to be developed. For low scale protein production (mainly for research purposes), intrinsic attributes of the protein are usually the most important parameter, but for large scale production (industrial scale) other criteria are also relevant. A summary of the factors that influence the host selection for low and industrial scale can be found in Table 2.

In either production scale, the intrinsic characteristics of the protein are of the utmost importance. These include the requirement for post-translational modifications such as glycosylation, disulfide bond formation, phosphorylation, acetylation, acylation, carboxylation and other modifications that can affect folding and activity [53]. Some of these modifications can be performed *in vitro*, namely in refolding strategies, but this is often not ideal. The production level is also very important in both cases because if a protein is inherently unstable in a given host, or if the host is for any reason incapable of expressing it in reasonable amounts, another system must be used. This may be related to the codon bias of the gene (which can be easily solved), but also to the instability of the produced mRNA, the susceptibility to attack by host proteases (protease deficient hosts are sometimes available), the incompatibility with secretion, or the deficient folding and aggregation in a particular host or cellular compartment. The technical expertise of the staff/researcher may also be an important factor. In the lab, prior experience with a certain system may introduce a certain bias to a particular host and in industrial settings, a small diversity of “cell factories” is often used in a given facility so that the staff becomes highly experienced in dealing with the peculiarities of each system.

The cell growth rate is important in both production scales, but it has a much larger impact in industrial production. If the cell growth is slow, the bioreactor must be operated during a longer period and this may affect the scheduling of the unit operations in a production plant. Since in some cases downstream processing equipment is shared by different production lines, an increased bioreactor operation time may impact on the possibility to integrate the production of that particular protein in the industrial unit which may be producing several products. The time for setup and development is also important in both production scales and it concerns the time required to go from gene to product. Since in industrial production, protein products often require approval by regulatory bodies (e.g., the Food and Drug Administration, FDA) and this approval process must follow the process development, this issue becomes critical in the industrial case and is somewhat less important at the lab scale. The scalability of the process is also important at both production levels, but has a more profound effect on industrial production. At the host level, scalability means that the cells will be able to withstand very demanding cultivation conditions, particularly concerning the sensitivity to shear forces, tolerance to deficient oxygen mixing and heat and mass transport limitations that are common in large scale reactors. Additionally, the possibility of performing fed-batch fermentation in HCDC mode is also an advantage of some of the hosts.

For large scale production and commercialization, issues like the safety component are also important. If possible, the organism should have a GRAS (Generally Regarded As Safe) status so that additional precautions are not necessary and even cheaper (e.g., non-explosion proof) equipment or handling procedures can be adopted [54]. The issues surrounding regulation are also critical as many protein products must be approved by regulatory bodies as mentioned before. The regulatory bodies will make sure that the protein product is safe and it can provide the claimed effect before commercialization. Hosts with a very solid track record of approval are most likely to get faster and favorable decisions from the regulatory bodies. If a protein product is under patent protection or even if the process of producing the desired protein is protected, the inventors may be entitled to royalties until the patent expires. Thus, it is important to guarantee that the product or the production process does not infringe pre-existing patents [55]. The cost of goods is particularly important in large scale production because of the amount of culture medium components and also other components required during downstream or formulation. Particular attention is given to the culture medium, which sometimes is very expensive due to the metabolic requirements of the host organism. However, in some cases, it can include wastes and by-products of other industries as a cost reduction strategy.

**Table 2.** Factors that influence the selection of the most appropriate expression host for low and industrial scale production.

Low level production	Industrial production
Intrinsic protein properties	Intrinsic protein properties
Level of production	Level of production
Technical expertise	Technical expertise
Cell growth	Cell growth
Time for setup and development	Time for setup and development
Scalability potential	Scalability potential
	Safety component
	Regulatory issues
	Patenting issues
	Cost of goods

For the purpose of this review, we are going to focus our attention on microbial systems since recombinant protein production in biofilms is being explored in this context. A closer look at the available literature shows that from the many microbial hosts that are currently used for recombinant protein production in the planktonic state, only a few have ever been tested for recombinant production in biofilms (Table 1). Table 1 shows only four microbial hosts, two bacteria and two filamentous fungi. The relative advantages and disadvantages of each system will be discussed below and summarized in Table 3.

### 4.1. Escherichia coli

The Gram-negative bacterium *E. coli* has been the most intensively used workhorse for recombinant protein production since the 1980s. The profound knowledge that has accumulated about this organism and the associated genetic methodologies still make it a highly versatile host capable of adaption to different production requirements [56]. *E. coli* grows fast (doubling times of 20 min can be achieved) in non-expensive culture media [3],[57],[58], and the fermentation is easy to scale-up. In this host, there is no unintended glycosylation in non-engineered strains, no viral contamination risk (with viruses that can affect humans) and it has a very solid track-record in product approvals by the FDA. Additionally, some strains have a GRAS status, it can accumulate recombinant proteins at more than 20% of the total cellular protein content, a very well-established fermentation know-how is available and it is amenable to HCDC [58]. Although there are many available expression strains, some of them have been specifically manipulated to be used with particular expression systems [3].

Protein production can be targeted to different locations (cytoplasm, periplasm, inner/outer membrane, or culture medium) with significant impact in downstream processing [59]. Although extracellular production can greatly reduce the complexity of the production process, the low secretion capacity of *E. coli* has always been considered as a major drawback. Different techniques can be used to circumvent this limitation such as the engineering of dedicated secretion systems existing in pathogenic strains, the use of native translocation mechanisms eventually using carrier-proteins, or cell envelope mutants or the co-expression of lysis-promoting proteins [4],[59].

The inability to perform glycosylation and several other post-translational modifications have also been a hallmark of *E. coli*. Although it was initially thought that bacteria were not capable of producing glycosylated proteins, the discovery of an N-linked glycosylation system in *Campylobacter jejuni* changed this perception. This system was transferred to *E. coli* [60] and the production of glycosylated proteins at a scale of 5 L was made possible [61]. There are still several significant challenges to be overcome in the production of glycosylated proteins in *E. coli*. Not only sometimes glycosylation is incomplete, but also its efficiency can be very low and heterogeneity can be found if more than one glycosylation site is present. Additionally, the production of glycosylated proteins was shown to negatively affect cell growth [4]. Since most of the eukaryotic proteins are N-terminally acetylated, this modification is very important in recombinant protein production in prokaryotic systems as acetylation has shown to affect several cellular processes. Also, phosphorylation is very important for the activity of many recombinant proteins and it was shown that either by co-expressing chaperone proteins or by performing gene fusions, it was possible to obtain some acetylated and phosphorylated proteins in *E. coli* [4].

Other drawbacks are the possibility of acetate accumulation, which has a toxic effect, although this can be controlled by the oxygen level and careful choice of the expression strain. Additionally, many proteins are produced as insoluble inclusion bodies, which are inactive and require refolding. Although it has been argued that recombinant protein production in inclusion bodies decreases the deleterious effects associated with protein toxicity (as the protein is not active), it must be remembered that high-level recombinant protein production is always toxic to the cells due to the metabolic burden associated with the expenditure of energy, amino acids and other precursor molecules. In order to obtain an active protein from these inclusion bodies, it is often necessary to remove them from the cells, the proteins must be solubilized by denaturants that cause protein unfolding, and disulfide bonds must be broken using reducing agents. Refolding than proceeds by removing the denaturant and reducing agent followed by renaturation with oxidation agents [5]. A further disadvantage of *E. coli* is the accumulation of lipopolysaccharides (LPS), requiring additional purification steps as some of these substances are pyrogenic in humans [62].

Another possible application of biofilms as a system to produce valuable proteins is the possibility of expressing membrane proteins. These play key roles in many diseases and about 70% of all drugs act on membrane proteins [63]. A particular type of membrane proteins (helical bundle type) is difficult to produce in inclusion bodies and must be produced in a way that the protein is inserted into the membrane from where it can be purified. This may be problematic because the capacity of the translocation machinery of an *E. coli* cell may be saturated, thus preventing efficient transport to the cell envelope [64],[65]. In order to circumvent this problem, different strains were developed for membrane protein expression in which expression rates can be tuned to match the capacity of secretion systems [66], and also omitting the inducer in chemically inducible promoter systems has been tested with success [67]. These systems are based on expressing the recombinant protein at a lower rate and since biofilm cells are usually at a lower metabolic state then their planktonic counterparts, it is tempting to use them for this purpose. On the other hand, biofilm formation depends on the production of EPS and the transport of these molecules to the cell exterior will take its share of the translocation capacity of the cell.

### 4.2. Bacillus subtilis

This organism is arguably the best studied Gram-positive bacterium [68] and has been intensively used for the production of different enzymes like proteases and amylases, mainly for detergents and the food industry. The production of these enzymes is also regulated, but criteria are less stringent than when producing recombinant proteins for therapeutic application. A major advantage of *B. subtilis* when compared to *E. coli* is that it does not have an LPS-containing outer membrane [5], which is important as LPS are pyrogenic in humans [62]. Several homologous proteins have been produced in this host [5], but sometimes the expression of heterologous proteins is more challenging [69],[70]. Several strains of *B.* *subtilis* have been sequenced and they not produce toxins [5]. *Bacillus* species are capable of fast growth on cheap carbon sources and are robust for use in industrial fermentations [71]. The secretion capacity of the system is high [72], which facilitates downstream processing. Several *Bacillus* strains produce different proteases [69], but some protease-deficient strains are available. This host is generally regarded as a potentially safe bacterium for industrial and pharmaceutical applications [68]. Plasmid instability can also be an issue when working with this organism, but it has been shown that this disadvantage can be overcome by using plasmids with theta replication [69],[72]. For recombinant protein production, *B. subtilis* is being intensively used and current development strategies include promoter engineering, signal peptide engineering to increase secretion efficiency and development of protease-defective strains [68]. Further knowledge must also be gathered about the physiological responses of producing strains during large-scale fermentation, and there is a lack of detailed knowledge on protein-protein interactions and the post-translational regulation network [71]. Operational challenges like the production of large amounts of foam, sporulation under nutrient starvation [70] and high maintenance metabolism are being addressed through genetic manipulation [71].

### 4.3. Aspergillus niger and A. oryzae

*A. niger* and *A. oryzae* are considered non-mycotoxin producers, although low levels of mycotoxins have been found in particular fermentation conditions [73]. Both species have a GRAS status due to long-term use without pathogenicity.

These are very attractive hosts for recombinant protein production since they can secrete high levels of active proteins with post-translational modifications such as glycosylation [5]. Additionally, it has been shown that some strains have a similar glycosylation pattern to that of human cells [54],[73] and that these species can grow at high rates and to high densities in commercial bioreactors [74].

Although the production of homologous proteins is sometimes successful, the production of heterologous proteins is often difficult [75],[76]. Secretion of some recombinant proteins is low in some cases, and production seems to be limited by transcriptional and post-translational metabolic steps [5],[54]. There is still limited knowledge about the details of protein secretion and modification in these organisms. Also, foreign DNA is sometimes quickly degraded and expression rates are often reduced due to rapid RNA degradation [76], or processing. Additionally, the glycosylation pattern affects folding and secretion, and therefore incorrectly glycosylated proteins are degraded [54]. Being saprophytic organisms, these organisms naturally secrete large amounts of proteases that degrade foreign proteins [73],[75] and cell productivity is highly affected by the growth morphology.

Despite the challenges posed by broth rheology when it comes to mass transfer, non-filamentous, less viscous, low-protease producing strains have been developed [5],[74]. *Aspergillus* species are good candidates for heterologous protein production and other foreign metabolites, thus acting as cell factories [77].

**Table 3.** Advantages and disadvantages of the different hosts used for recombinant protein production in biofilms.

Host	Advantages	Disadvantages
*Escherichia coli*	Proven track record in FDA approvals Many available expression systems Simple, well-understood genetics Inexpensive culture media Fermentation easy to scale up No unintended glycosylation No viral or prion contamination risk Ease of handling, GRAS status Fast proliferation High expression levels Very well-established fermentation know-how Amenable to HCDC Protein can be directed to different cellular locations	Proteins with disulfide bonds are difficult to express Production of glycosylated proteins is challenging Phosphorylated or acetylated proteins are difficult to produce Proteins produced with endotoxins Acetate formation resulting in cell toxicity Proteins produced as inclusion bodies Inactive proteins require refolding Protein stability can be low due to proteolytic degradation Protein secretion is challenging
*Bacillus subtilis*	Strong secretion capacity Ease of genetic manipulation Genetically well-characterized systems Superior growth characteristics Metabolically robust GRAS status No production of toxins Outer membrane devoid of LPS Fast growth on cheap medium	Protein instability due to host proteases Sporulation can be an issue Excessive foaming High maintenance metabolism
*Aspergillus niger* and *A.* *oryzae*	Enormous nutritional flexibility Able to perform complex post-translational modifications Glycosylation pattern may be similar to mammalian cells Some strains have a GRAS status Very high secretion potential HCDC is possible	Secretion levels are sometimes low Production can be limited by transcription Some strains encode many proteases Possibility of obtaining a highly viscous culture broth mRNA stability can be an issue Plasmid stability may be an issue Lack of knowledge about physiology

*Abbreviations:* FDA – Food and Drug Administration; GRAS – Generally Regarded As Safe; HCDC – high cell density cultivation; LPS – lipopolysaccharides.

## 5. Conclusions

Biofilm reactors present many advantages over suspended cell reactors, including higher cell densities and long-term stability as required for continuous processing. Furthermore, according to the work published on recombinant protein production, microbial biofilm systems are sometimes able to produce recombinant proteins at attractive levels. Nevertheless, several elements that affect the overall efficiency of biofilm reactors, especially limitations in the diffusion of nutrients and oxygen, call for careful implementation of strategies specifically targeted for increasing recombinant protein production in these systems.

Additionally, there are several hosts that can be chosen for recombinant protein production in biofilms. However, the selection of the most appropriate expression host is limited to a few options given the characteristics of the protein and the process to be developed. In this review, we focused on two bacteria (*E. coli* and *B. subtilis*) and two filamentous fungi (*A. niger* and *A. oryzae*) since they have already been tested for recombinant production in biofilms. *E. coli* remains one the favorite hosts for recombinant protein production at lab and industrial scale since it offers many advantages over the other host systems, namely fast growth at high cell densities, well-characterized genetics, and availability of a large number of cloning vectors.
